# Prevalence of Irritable Bowel Syndrome in Saudi Arabia: A Systematic Review and Meta-Analysis

**DOI:** 10.7759/cureus.45357

**Published:** 2023-09-16

**Authors:** Manal Almasary, Khalid M Alkhalifah, Solaf Hilal Alotaibi, Mohamed Elhefny, Haila Alabssi, Sara Saeed Alaklabi, Rasil Sulaiman Alayed, Anwar A Alghamdi, Abdulmajeed Albalawi

**Affiliations:** 1 Internal Medicine, Umm Al-Qura University, Al Qunfudhah, SAU; 2 Unaizah College of Medicine and Medical Sciences, Qassim University, Buraydah, SAU; 3 College of Medicine, King Saud Bin Abdulaziz University for Health Sciences, Jeddah, SAU; 4 Medical Genetics, Umm Al-Qura University, Al Qunfudhah, SAU; 5 Family and Community Medicine, Imam Abdulrahman Bin Faisal University, Dammam, SAU; 6 Internal Medicine, Bisha University, Bisha, SAU; 7 College of Medicine, Qassim University, Buraydah, SAU; 8 Faculty of Medicine, Al-Baha University, Al-Bahah, SAU; 9 Radiology Department, Maternity and Children's Hospital, Tabuk, SAU

**Keywords:** systematic literature review, ibs, meta-analysis, irritable bowel syndrome, saudi, prevalence

## Abstract

Irritable bowel syndrome (IBS) is a commonly found global functional pathology with no detectable structural changes. It affects the quality of life and often coexists with psychiatric and somatic issues. We reviewed all articles published between 1990 and April 2023. The databases used for the data search were Google Scholar, Web of Science, Cochrane Library, and PubMed/MEDLINE. Ovid search engine was also used to broaden the search strategy. Predefined keywords, including "Irritable bowel syndrome" (MeSH) OR "IBS" (all fields), along with "Saudi Arabia" and "Middle East," were used to avoid data loss and ambiguity. Any cross-sectional study that reported the prevalence of IBS in any gender, age, and group of the Saudi population was included. Data extraction was independently performed in duplicate to mitigate bias and data loss. Statistical analysis of proportion was conducted by using Miller (Freeman-Tukey double arcsine - exact inverse). Out of 350 records identified, only 38 studies were included in the quantitative synthesis. The total number of study participants was 26,567, on the basis of the predefined inclusion criteria of the study. StatsDirect software was used for the statistical analysis of the study parameters. Based on all 38 identified studies, the calculated pooled prevalence was 20.7% (95% confidence interval (CI) = 17.8% to 23.7% by applying random effects (DerSimonian-Laird method). IBS was identified in 21% (95% CI = 16.7% to 25.7%) of the general population, 22% (95% CI = 17.6% to 26.7%) of students, and 18.3% (95% CI = 13.3% to 23.9%) of healthcare workers. The pooled prevalence of IBS among the Saudi population was 20.7%. The pathophysiology of IBS is complexed and significantly affected by genetics, diet, cultural characteristics, age, anxiety, depression, stress, and sleep disorders. This study fills a gap in understanding IBS prevalence in Saudi Arabia, contributing valuable data to this region.

## Introduction and background

Irritable bowel syndrome (IBS) is a functioning bowel problem that is prevalent [1]. It does not happen by structural or metabolic alterations that are detected by conventional diagnostic techniques [1]. However, the pathophysiology behind IBS is yet not completely understood and suggested as multifactorial pathology. The latest studies suggest that it is a brain-gut disorder, where the symptoms are caused by a complicated connection between the gastrointestinal (GI) and central nervous systems [2]. IBS can manifest as bloating, irregular stools, and abdominal discomfort. Specific individuals may experience severe symptoms, whereas others may experience moderate or mild symptoms. In addition, IBS frequently occurs alongside some psychiatric disorders, such as anxiety and depression. It also occurs alongside multiple somatic abnormalities, such as pain syndromes, overactive bladder, and migraines. Moreover, IBS is considered one of the pathologies linked with poor quality of life [1]. An estimated 10% of the overall population worldwide is found to have IBS. Unfortunately, in some countries, there are no data available regarding IBS prevalence. However, in Southeast Asia and the Middle East, the prevalence is around 7%; in North America, North Europe, and Australia, it is around 11.8-14%. Moreover, in South Europe, Africa, and South America, it is around 15-21% [3].

IBS is a disorder that can be difficult to diagnose due to its changing symptoms, similarity to other disorders, the lack of a precise biomarker, and the absence of a single test with perfect sensitivity and specificity. As a result, IBS is diagnosed based on a combination of symptoms, signs, and laboratory investigations. Over the years, different diagnostic guidelines have been established, encompassing the Kruis scoring system and Manning and Rome criteria [4]. The Rome I, II, and III criteria are widely used for IBS diagnosis. Each of these criteria has strengths and weaknesses, and the Rome III criteria are currently the most widely used. In 2016, the Rome IV criteria were introduced, incorporating several modifications compared to the Rome III criteria. These changes encompassed eliminating "discomfort" from the description, a heightened emphasis on the frequency of abdominal pain, acknowledgment of bloating and distention as prevalent indications, and a more precise definition of irregular bowel patterns. The Rome IV criteria additionally make it clear that the prevailing bowel patterns determine IBS subtypes during days with atypical bowel movements. In general, comprehending the progression of diagnostic standards for IBS is crucial for healthcare professionals to diagnose and address this condition accurately [4]. IBS is linked to high healthcare costs, psychological difficulties, and a substantial disease burden.

The quality of life of IBS patients is markedly poor, and several factors have been identified as predictors of low generic quality of life, including GI manifestations; psychological symptoms, such as depression, anxiety, stress, GI-symptom specific anxiety (GSA), and non-GI somatic signs; and demographic characteristics, such as gender and age. While the severity of GI symptoms holds significance, its impact on the quality of life seems relatively minor compared to other more influential factors. Essential among these is GI-specific anxiety, which is a primary determinant of the quality of life of individuals with IBS. This anxiety might also play a role in connecting the effects of other recognized elements on the mental and physical well-being. Recognizing and comprehending these factors are pivotal in effectively addressing and managing the better quality of life for patients living with IBS [5]. The study was formulated for the accurate assessment of IBS prevalence among Saudi population. Understanding its prevalence in the Saudi population is essential for healthcare planning and resource allocation, as it can help identify the scale of the problem and guide interventions.

## Review

Methods

Strategy of Scientific Database Search

The research panel designated two authors to conduct a comprehensive search of data from all leading scientific databases individually. The databases identified for the advanced data search were Google Scholar, Cochrane Library, Web of Science, and PubMed/MEDLINE. The research team also included Ovid search engine to carry out an inclusive search. All cross-sectional studies were filtered and included. Other filters, including patient’s age, gender, and population categories, were not imposed. The keywords of the data search were predefined to avoid any data loss and ambiguity. The keywords used were "Irritable bowel syndrome"(MeSH) OR “IBS” (all fields) with "Saudi Arabia" and "Middle East." Data were extracted from 1990 to April 2023. The data were analyzed thoroughly by both the assigned authors, in case any extracted record is missed.

Selection Criteria of Extracted Records

Inclusion criteria of records: Any scientific study reported the prevalence of IBS in any gender, age, and Saudi population group from 1990 to April 2023. Both authors performed study selection twice individually to avoid data bias. All cross-sectional studies from all population categories were facilitated to conduct this study.

Exclusion criteria of records: Any non-scientific, incomplete, non-conclusive conference abstract/poster, review, letter to the editor, case report, and non-English studies were excluded.

Primary Outcome Measures of the Study

The estimation of the IBS prevalence in the Saudi population was established.

Selection of Studies

All extracted data were screened and selected based on the inclusion criteria by both authors independently. The research team finalized the selected studies and resolved any ambiguity by consensus.

Data Extraction

Data extraction was performed in a duplicate manner independently to avoid any data loss and risk of bias [6].

Methodology Statement and Data Presentation

**Figure 1:**
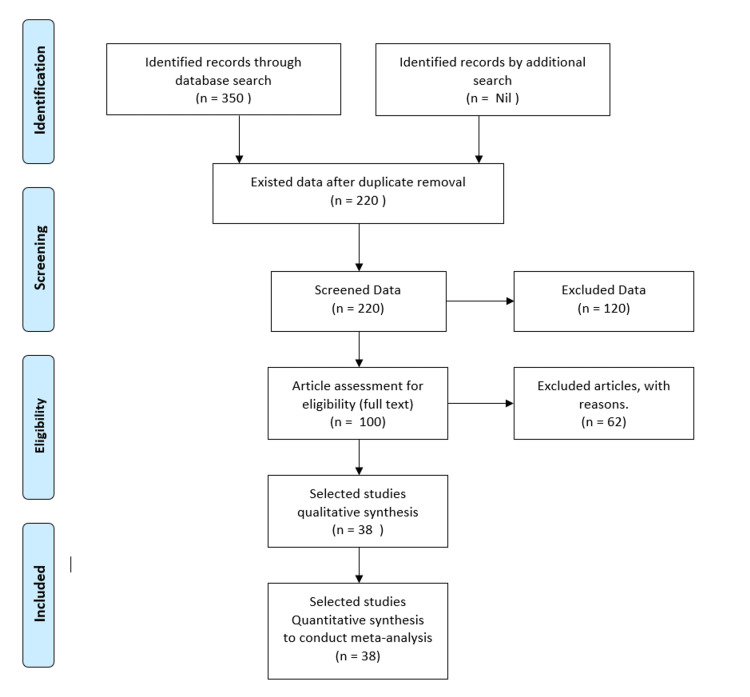
Flowchart of the study selection process.

The data were extracted and screened, as per the inclusion criteria, and thoroughly analyzed for eligibility and selection. The Preferred Reporting Items for Systematic Reviews and Meta-Analyses (PRISMA) was adapted for the methodology statement and data presentation [7], as illustrated in Figure 1, to conduct the systematic review and meta-analysis (SR & MA).

 A comprehensive review of all the identified and chosen studies is outlined in Table 1.

**Table 1:** Characteristics of the included studies. IBS: irritable bowel syndrome

S. No.	First author, year, and reference no.	Study type	Duration of study	Study population	Gender	Diagnostic criteria	Total no. of study population	IBS prevalence (%)
1	Alshahrani, 2020 [8]	Cross-sectional study	February 2020	Male secondary school Saudi students in Najran City	Male	Rome IV criteria	400	39.80%
2	Arishi, 2020 [9]	Cross-sectional study	January-March 2020	Adult general population	Both	Rome IV criteria	1,554	16%
3	Almezani, 2018 [10]	Cross-sectional study	February-March 2018	Medical students at the University of Hail	Both	Rome IV criteria	133	28.50%
4	Al-Zahrani, 2022 [11]	Cross-sectional study	October-November 2021	Umm Al-Qura University medical students	Both	Rome III criteria	303	33%
5	Alnasser, 2022 [12]	Cross-sectional study	November 2022	Adult general population	Both		279	17.6%
6	Alhazmi, 2009 [13]	Cross-sectional study	April 2009	Secondary school male students	Male	Manning and Rome II criteria	1,747	9.20%
7	Aljammaz, 2020 [14]	Cross-sectional study	March-May 2019	General population	Both	Rome III criteria	426	30.5%
8	Alanazi, 2021 [15]	Cross-sectional study	April-December 2019	High school female students	Female	Rome IV criteria	230	54.8%
9	Hanan, 2016 [16]	Cross-sectional study	December 2013-May 2014	Umm Al-Qura female students in health-related faculties	Female	Rome III criteria	1,351	33.7%
10	Taha, 2019 [17]	Cross-sectional study	_	Healthcare workers at the primary healthcare (PHC)	Both	Rome IV criteria	205	16.1%
11	Hussein, 2021 [18]	Cross-sectional study	November-February 2020	Adult general population	Both	Rome IV criteria	104	7.9%
12	Alharbi, 2022 [19]	Cross-sectional study	February-April 2022	General population of Makkah City	Both	Rome IV criteria	921	20.19%
13	Sibyani, 2018 [20]	Cross-sectional study	_	Healthcare workers at the PHC	Both	Rome IV criteria	190	28.4%
14	Alaqeel, 2017 [21]	Cross-sectional study	One year	Medical students	Both	Rome III criteria	270	21%
15	Hasosah, 2017 [22]	Cross-sectional study	Two months	Medical students	Both	Rome IV criteria	179	15.64%
16	Alqahtani, 2019 [23]	Cross-sectional study	Six months	General population	Both	Rome IV criteria	1,680	18.2%
17	Zarnoog, 2012 [24]	Cross-sectional study	10 months	Medical students	Both	Questionnaire developed by the World Gastroenterology Organization (WGO)	389	12.60%
18	Ibrahim, 2013 [25]	Cross-sectional study	2011-2012	Medical students	Both	Rome III criteria	597	31.8%
19	Ibrahim, 2016 [26]	Cross-sectional study	2014-2015	Nurses	Both	Rome III criteria	229	14.4%
20	Ibrahim, 2018 [27]	Cross-sectional study	2016-2017	Paramedical students	Both	Rome III criteria	525	33.3%
21	AlButaysh ,2020 [28]	Cross-sectional study	February-June 2018	Undergraduate students	Both	Rome IV criteria	767	15.8%
22	Alfaqih, 2022 [29]	Cross-sectional study	November-December 2020	Medical students	Both	Rome IV criteria	290	14.8%
23	Hakami, 2019 [30]	Cross-sectional study	2017-2018	University students	Both	Rome IV criteria	890	8.8%
24	Aldawsari, 2017 [31]	Cross-sectional study	-	Male medical students	Male	-	115	7.30%
25	Ahmed, 2020 [32]	Cross-sectional study	October 2019-January 2020	Medical students	Both	Rome IV criteria	472	12.6%
26	Al-bukhari, 2016 [33]	Cross-sectional study	May 2014	Medical students	Both		555	10.5%
27	Alharbi, 2019 [34]	Cross-sectional study	October 2018-February 2019	General population	Both	Rome IV criteria	920	11.8%
28	Alzahrani, 2018 [35]	Cross-sectional study	_	Medical students	Both	Rome IV criteria	856	17.5%
29	AlAmeel, 2019 [36]	Cross-sectional study	May-June 2018	Medical doctors	Both	Rome IV criteria	594	16.3%
30	Sachithananthan, 2018 [37]	Cross-sectional study	_	General population	Both		150	53.3%
31	Mohamed, 2016 [38]	Cross-sectional study	May 2016-June 2016	University students	Both	Rome lll criteria	228	32.5%
32	Basharat, 2022 [39]	Cross-sectional study	June 2022-November 2022	General population	Both	Rome IV criteria	6,300	23.81%
33	Alsuwailm, 2017 [40]	Cross-sectional study	August 2015-September 2016	Medical students	Both	Rome lll criteria	173	44.5%
34	Alharthi, 2022 [41]	Cross-sectional study	June 2021-August 2021	Female secondary school students	Female	Rome IV criteria	401	21.4%
35	Al-Turki, 2011 [42]	Cross-sectional study	January 2010-November 2010	University students	Both	Rome lll criteria	1,237	14.20%
36	Mirghani, 2022 [43]	Cross-sectional study	June-July 2021	Medical students	Both	Rome III criteria	215	22.80%
37	Wani, 2020 [44]	Cross-sectional study	January-March 2016	University students	Both	_	181	29.28%
38	Almutairi, 2017 [45]	Cross-sectional study	April-May 2016	Medical students	Both	Rome lll criteria	511	13.70%

Statistical Analysis

StatsDirect version 3.0 statistical analysis software (StatsDirect.exe; https://www.statsdirect.com/) was used for the statistical analysis of the study parameters. The statistical analysis of the proportion was conducted using Miller (Freeman-Tukey double arcsine - exact inverse).

Results

For the pooled proportion of IBS in all Saudi population of 38 identified studies, the DerSimonian-Laird method was applied. The scored pooled proportion by the random effects model was 0.207288 with 95% confidence interval (CI) = 0.1787 to 0.237398. The applied publication bias indicators were Egger, Harbord, and Begg and Mazumdar tests. The analyzed publication bias was 3.306361 (95% CI = -0.358925 to 6.971647) with P = 0.0756; 1.120381 (92.5% CI = -2.579007 to 4.81977) with P = 0.5821; and 0.297297 with P = 0.0083 for Egger, Harbord, and Begg and Mazumdar tests, respectively.

**Figure 2:**
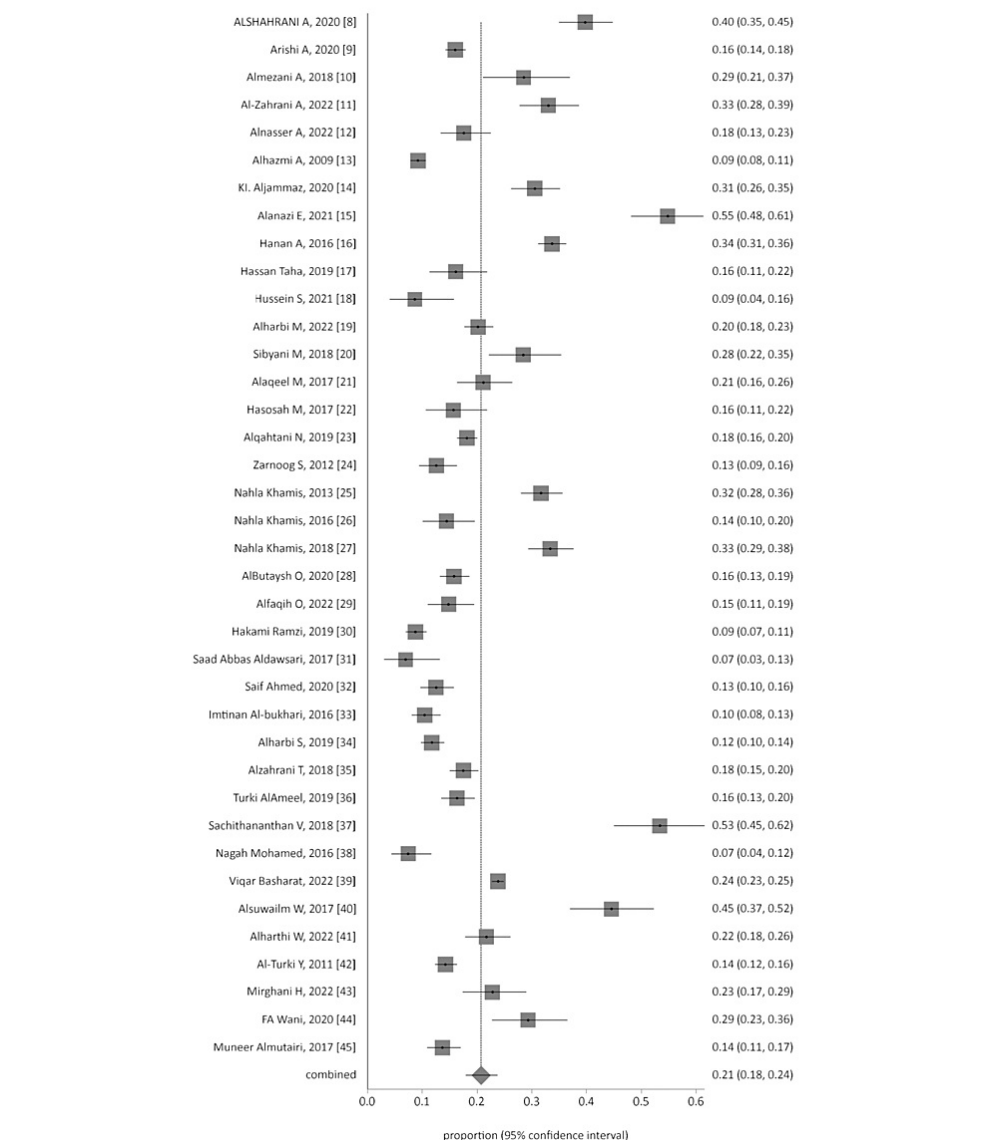
Proportion meta-analysis forest plot of all included studies.

**Figure 3:**
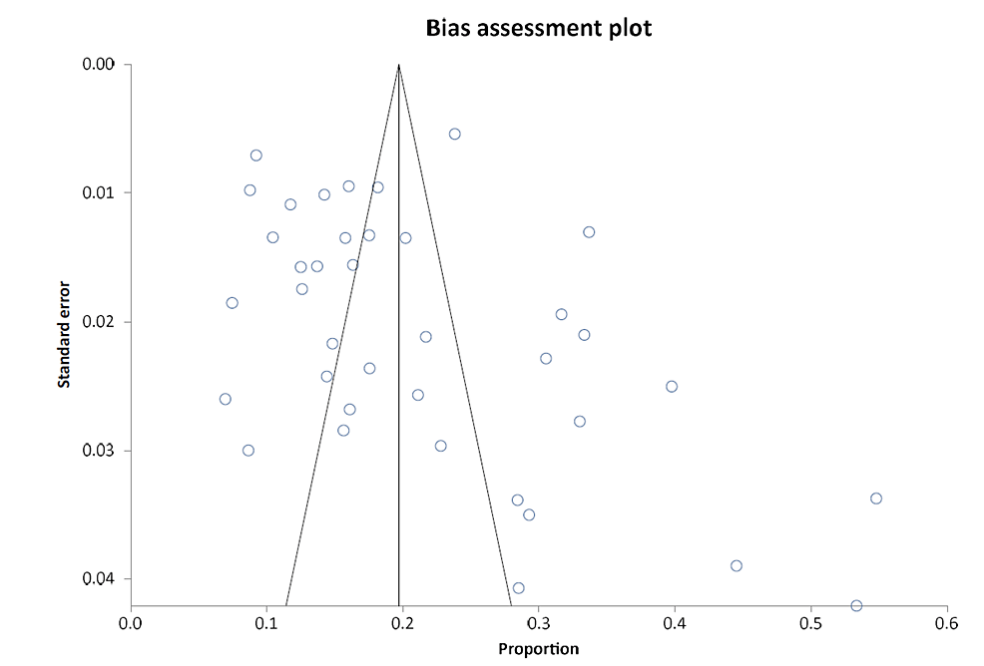
Funnel plot for the publication bias of all studies.

A forest plot was generated through a cumulative analysis of all identified studies of MAs (Figure 2). A funnel plot was designed to evaluate the publication bias of all studies, as presented in Figure 3.

Subgroup Analysis

**Figure 4:**
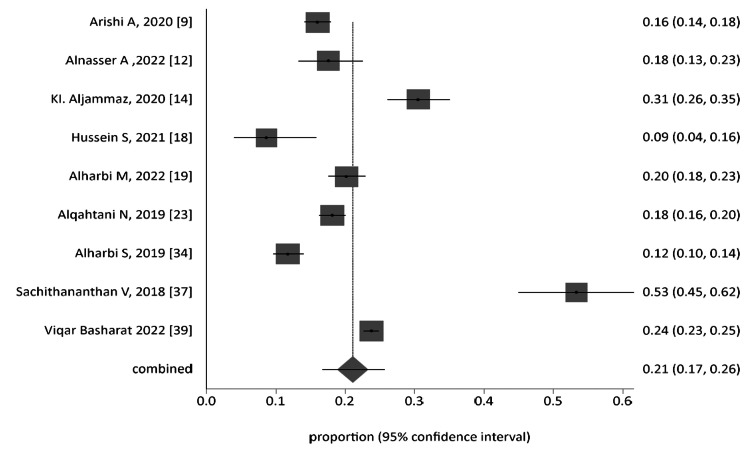
Proportion meta-analysis forest plot of the general Saudi population.

**Figure 5:**
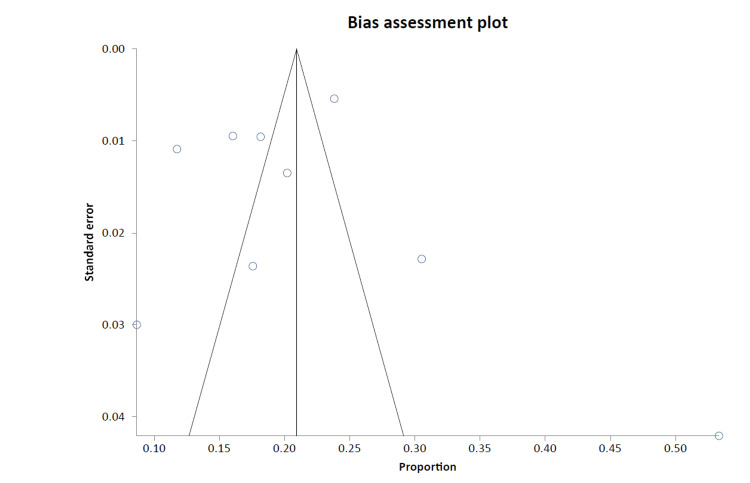
Funnel plot for the publication bias of the general Saudi population studies.

**Figure 6:**
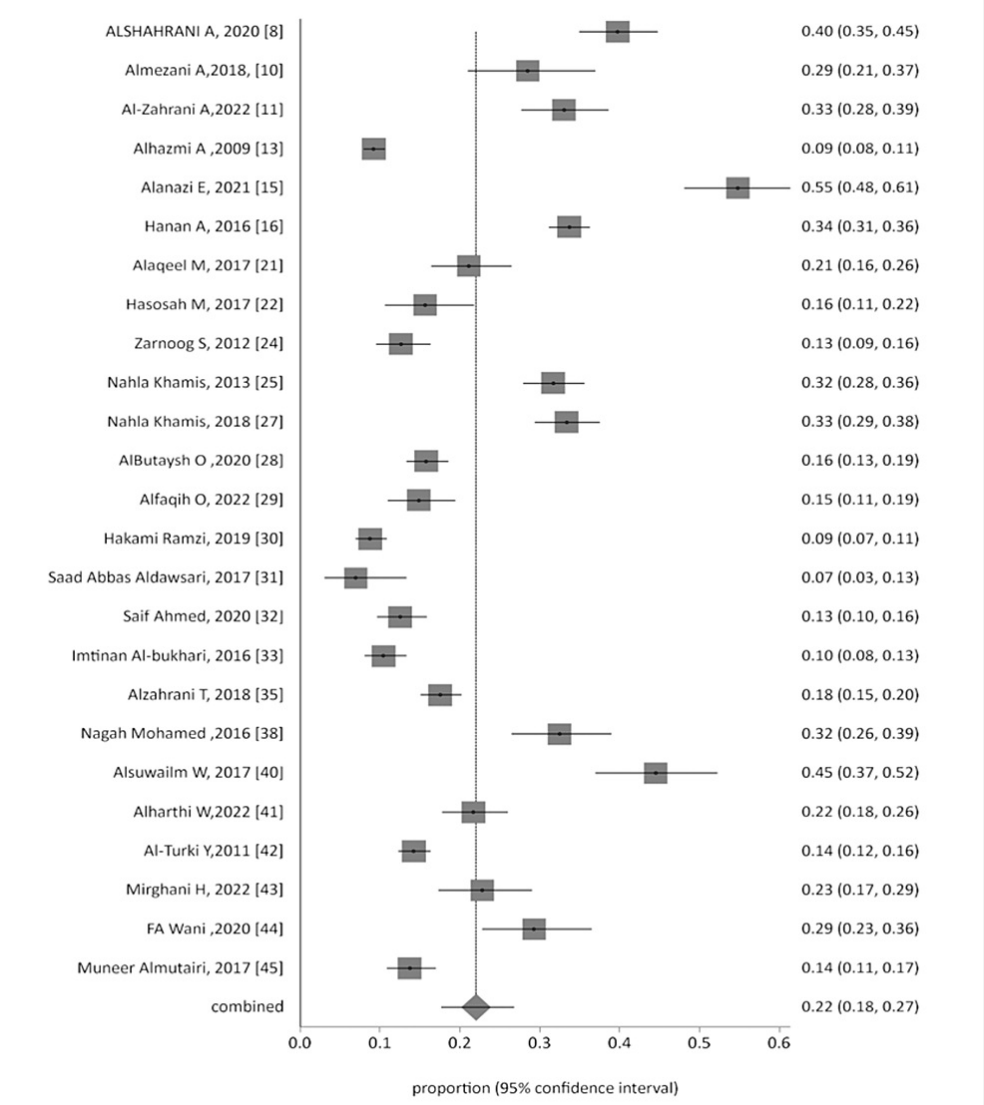
Proportion meta-analysis forest plot of the student group.

**Figure 7:**
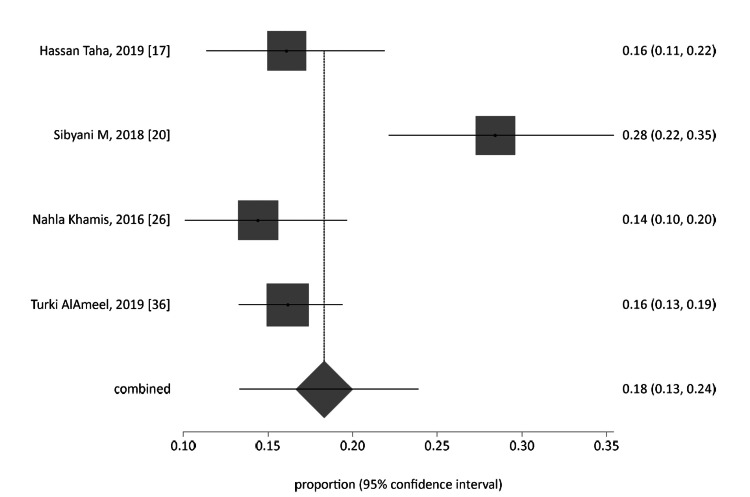
Proportion meta-analysis forest plot of healthcare professionals.

All 38 studies were categorized and analyzed into three subgroups: general population group, all student-based studies (from school, college, and universities), and workers of the health sector. The forest plot of each group was generated, as represented in Figure 4, Figure 6, and Figure 7 for the general Saudi population, students, and healthcare workers, respectively. The funnel plot for the publication bias of the general Saudi population studies is displayed in Figure 5.

The subgroup pooled proportion was 0.20902, with 95% CI = 0.201861 to 0.216271 by inverse variance using fixed effects. The heterogeneity of studies was evaluated by Cochran's Q test, which was calculated as 226.438368 (df = 8), P < 0.0001. The estimated variance between studies was 0.025593, I_2_ = 96.5% (95% CI = 95.3% to 97.2%). The subgroup pooled proportion was 0.21097 (95% CI = 0.167876 to 0.257535) by the DerSimonian-Laird method using random effects.

The analyzed publication bias was -0.245242 (95% CI = -8.872626 to 8.382141) with P = 0.9483; 0.418438 (92.5% CI = -7.939084 to 7.102209) with P = 0.9107; and 0.111111 with P = 0.7614 for Egger, Harbord, and Begg and Mazumdar tests, respectively. The student group is represented in Figure 6. The fixed-effect inverse variance was applied to calculate the pooled proportion. The calculated proportion was 0.192241 (95% CI = 0.185485 to 0.199087). The heterogeneity among the studies was 947.953475 (df = 24), P < 0.0001, using Cochran's Q test; the variance between the analyzed studies was 0.075817, I_2_ = 97.5% (95% CI = 97.1% to 97.8%). The DerSimonian and Laird method was applied, using random effects to obtain the pooled proportion, i.e., 0.220167 (95% CI = 0.176109 to 0.267617). Same-publication bias analysis was applied on this group, i.e., Egger’s test, Harbord bias, and Begg and Mazumdar test reported 8.604631 (95% CI = 4.255082 to 12.95418) with P = 0.0004; 6.295287 (92.5% CI = -0.334208 to 12.924783) with P = 0.0898; and 0.38 with P = 0.0073, respectively.

The healthcare professional group is represented in Figure 7. The calculated pooled proportion was 0.176021 (95% CI = 0.155024 to 0.198037) by applying fixed effects (inverse variance); I_2_ (inconsistency) = 80.6% (95% CI = 21.3% to 90.8%). The pooled proportion by random effects was 0.183642 (95% CI = 0.133978 to 0.239083). The evaluated Egger bias was 3.669959 (95% CI = -11.635854 to 18.975771), with P = 0.4107.

Data Findings

The findings of this SR & MA based on 38 studies, identified through the PRISMA guidelines, and the summary of all selected studies along with essential findings are presented in Table 1.

The 26,567 cumulative study participants were used to calculate the pooled prevalence of IBS, i.e., 20.7% (95% CI = 17.8% to 23.7%) by applying random effects (DerSimonian-Laird method).

The studies were further categorized into subgroups for the analysis of variation among different participant categories. The categorized groups were the general Saudi population, students (schools, colleges, and universities), and healthcare workers. The pooled prevalence among the three groups was 21% (95% CI = 16.7% to 25.7%), 22% (95% CI 17.6% to 26.7%), and 18.3% (95% CI = 13.3% to 23.9%) among the general population, students, and healthcare workers, respectively, using random effects (DerSimonian-Laird method).

Discussion

IBS is an important and neglected pathology, and its prevalence in the Saudi population is unknown. This SR & MA, based on 38 studies from all disciplines, reported the pooled prevalence of IBS among the Saudi population. A study by Alosaimi et al. reported that the IBS prevalence in Arab countries ranged from 8.9% to 31.8% [46]. Another systematic study published in 2020 reported that the global prevalence of IBS from 38 countries based on Rome III or Rome IV criteria was 9.2% and 3.8%, respectively, with women predominance [47]. However, the prevalence substantially varied between countries worldwide, and the reason still needs to be identified [2]. A 2023 study reported that the prevalence from Japan, China, and South Korea was reported as 14.9%, 5.5%, and 15.6%, respectively, with a 54.9% male population [48]. An SR by Al-baghli et al. based on Saudi studies reported that the IBS prevalence ranged from 7.9% to 40.7% [49]. The pooled prevalence of IBS among the Saudi population, identified in our study from all age groups, gender, and fields, was 20.7% (95% CI = 17.8% to 23.7%). Our findings are comparable to the global prevalence, as it varies from region to region. The 38 studies were based on both-gender, male-only, and female-only studies. Nevertheless, the gender predominance is still not concluded [47,48]; however, a higher prevalence was reported for females [3].

The study population was based on all fields, including the general population, medical college participants, university students, high school students, and healthcare professionals, including doctors and nurses. Most studies used Rome III and IV diagnostic criteria. IBS is a complex and deficiently understood disorder; apart from gastroenteritis, several risk factors are associated with this disorder, such as genetics, diet, cultural characteristics, age, anxiety, depression, stress, and sleep disorders [50,51].

We categorized our data into four groups to evaluate the variation of IBS prevalence: all studies, the general population, students, and healthcare professionals. The general Saudi population reported a pooled prevalence of IBS of 21% based on nine studies and 12,334 participants. Three studies, out of the nine, reported female predominance [9,12,14,18]. However, the associated risk factors were anxiety, depression, minimal physical activity, and less water intake [14,34]. The student population was the leading group of this SR & MA based on 25 studies among the 38 studies with a pooled prevalence of 22% from 13,015 study participants. Two studies from the student group reported male predominance [11,44], similar to the findings of Japan, China, and South Korea [3]. However, most studies reported female predominance [15,25,27]. The associated factors among these studies were poor diet, physical inactivity, depression, low quality of life, family history, and smoking [16,21,30,32,40,41]. Only four studies with 1,218 participants were based on healthcare professionals of both genders, with a pooled prevalence of 18.3%. The prevalence of IBS in the Saudi population is high comparable to the global prevalence, which shows the variability among counties globally. However, it is comparable to other Saudi studies [47,52].

Another factor of this prevalence is that most studies (65.7%) belonged to the student group. An SR based on Egyptian medical students reported an IBS prevalence ranging from 9.3% to 35.5%. The leading factor of the high prevalence in students was the stressful environment despite female gender, family history, psychiatric disorder, depression, anxiety, infection, dietary habits, and quality and pattern of sleep [53]. An SR & MA of university students from China, based on 22 cross-sectional studies, reported that the pooled prevalence of IBS was 11.89%. The leading associated factors in this study were gender, anxiety, depression, smoking, and drinking [54]. However, we found a much higher prevalence in our student population. Bashir et al. reported a cross-sectional study in 2022 on 300 Saudi medical students and reported a 49.3% IBS prevalence in undergraduate medical students, which is alarming. This study found a double likelihood of the female gender having IBS. Stress and poor lifestyle habits were strongly associated with this high prevalence [55].

Stress and a sedentary lifestyle are strongly linked to high IBS prevalence [55,56]. IBS is a gut-brain disorder that presumptively links to anxiety and depression [57]. Medical professionals' and students' lifestyle is a stressful domain. An MA based on data from seven countries reported a 16% prevalence of IBS among medical staff, which is also comparable to our findings [58]. We did not find significant differences in IBS prevalence among our study's different groups. However, among all the studies, the highest prevalence, i.e., 44%, was reported by Alsuwailm et al. using Rome III criteria in medical students, and the lowest prevalence was observed in the adult general population group, i.e., 7.9%, based on Rome IV criteria [18,40]. Nineteen studies used Rome IV diagnostic criteria, which is said to be more specific and accurate concerning positive probability [59], whereas 12 studies used Rome III criteria. Thirty-two studies were based on both genders. Eight reported predominance in females, and four studies reported higher prevalence among males, which is nonconclusive.

However, most global studies reported female predominance, and it is believed that sex hormones might promote gender differences by influencing stress response, gut-brain interaction, immune response, gut microbiome, and intestinal barrier functionality [60]. There was marked heterogeneity reported among the prevalence rate of different countries globally.

The limitations of our study is the marked heterogeneity seen among the studies based on Cochran's Q test, variance between the analyzed studies and I_2_ test, which indicates larger variation among selected studies.

Our study is an essential addition to the literature as no SRs and MAs in Saudi Arabia were conducted on the similar topic.

## Conclusions

We formulated this SR & MA to ascertain the prevalence of IBS in Saudi Arabia. The pooled prevalence of IBS among the Saudi population was 20.7%. This result is consistent with global trends, where IBS prevalence varies regionally. The pathophysiology of IBS is complex and significantly affected by genetics, diet, cultural characteristics, age, anxiety, depression, stress, and sleep disorders. This study fills a gap in understanding IBS prevalence in Saudi Arabia, contributing valuable data to this region.

## References

[1] Enck P, Aziz Q, Barbara G (2016). Irritable bowel syndrome. Nat Rev Dis Primers.

[2] Drossman DA (2016). Functional gastrointestinal disorders: history, pathophysiology, clinical features and Rome iv. Gastroenterology.

[3] Lovell RM, Ford AC (2012). Global prevalence of and risk factors for irritable bowel syndrome: a meta-analysis. Clin Gastroenterol Hepatol.

[4] Lacy BE, Patel NK (2017). Rome criteria and a diagnostic approach to irritable bowel syndrome. J Clin Med.

[5] Trindade IA, Melchior C, Törnblom H, Simrén M (2022). Quality of life in irritable bowel syndrome: exploring mediating factors through structural equation modelling. J Psychosom Res.

[6] Higgins JP, Altman DG, Gøtzsche PC (2011). The Cochrane Collaboration's tool for assessing risk of bias in randomised trials. BMJ.

[7] Swartz MK (2011). The PRISMA statement: a guideline for systematic reviews and meta-analyses. J Pediatr Health Care.

[8] Alsahrani AS (2020). Prevalence and risk factors for irritable bowel syndrome among male secondary school Saudi students in Najran City, Saudi Arabia. Med J Cairo Univ.

[9] Arishi AM, Elmakki EE, Hakami OM (2021). Irritable bowel syndrome: prevalence and risk factors in Jazan Region, Saudi Arabia. Cureus.

[10] Almezani A, Alkhalaf A, Alharbi M (2018). Prevalence of irritable bowel syndrome among medical students in Hail University, Saudi Arabia. Egypt J Hosp Med.

[11] Al-Zahrani A, Al-Shanbari A, Al-Sulami A, Zamzami O, Sindy A, Albagami S, Shatla M (2022). Prevalence of irritable bowel syndrome and its association with academic performance in Umm Al-Qura University medical students. Med Sc.

[12] Alnasser AH, Al Kalif MS, Alrowaila MA (2023). The irritable bowel syndrome among adults in Qatif, Saudi Arabia: prevalence and impact on health-related quality of life, by gender and age [version 1; peer review: 1 approved with reservations]. F1000Research.

[13] Alhazmi AH (2011). Irritable bowel syndrome in secondary school male students in AlJouf Province, north of Saudi Arabia. J Pak Med Assoc.

[14] Aljammaz KI, Alrashed AA, Alzwaid AA (2020). Irritable bowel syndrome: epidemiology and risk factors in the adult Saudi population of the central region. Niger J Clin Pract.

[15] Alanazi EO, Alshammri WO, Hammad SM, Mohammed AE (2021). Prevalence and risk factors for irritable bowel syndrome among high school female students in northern borders region, Saudi Arabia. Med Sci.

[16] Ali HS, Ibrahim Y, Saati AA, Esam-Eldin E, Al Harbi M (2016). Prevalence of irritable bowel syndrome and its relation to self-esteem, depression, and quality of life of female students in health-related faculties at Umm Al-Qura University. Am J Sci.

[17] Taha HY, Al Johani A (2019). Prevalence and associated factors of irritable bowel syndrome among healthcare professionals in primary health care setting in Al-Madinah, Saudi Arabia. Malaysian J Public Health Med.

[18] Amin HS, Irfan F, Karim SI (2021). The prevalence of irritable bowel syndrome among Saudi population in Riyadh by use of Rome IV criteria and self-reported dietary restriction. Saudi J Gastroenterol.

[19] Alharbi MH, Alhazmi AH, Ujaimi MH, Alsarei M, Alafifi MM, Baalaraj FS, Shatla M (2022). The prevalence of irritable bowel syndrome and its relation to psychiatric disorders among citizens of Makkah Region, Saudi Arabia. Cureus.

[20] Sibyani MJ, Bardisi W, Ibrahim A (2017). The prevalence of irritable bowel syndrome and its associated factors among physicians working at primary health care centers of ministry of health in Jeddah, Saudi Arabia. Int Ann Med.

[21] Alaqeel MK, Alowaimer NA, Alonezan AF, Almegbel NY, Alaujan FY (2017). Prevalence of irritable bowel syndrome and its association with anxiety among medical students at King Saud bin Abdulaziz University for Health Sciences in Riyadh. Pak J Med Sci.

[22] Hasosah M, Alamri S, Al-Husayni F, Aljedaani R, Zwawy M, Al-Zahrani A (2017). Prevalence of irritable bowel syndrome among medical students and interns in Jeddah, Saudi Arabia. J Clin Med Case Stud.

[23] Alqahtani NH, Mahfouz ME (2022). The prevalence and risk factors of irritable bowel syndrome in Saudi Arabia in 2019. Int J Prev Med.

[24] Zarnoog SS, Al-Omrani AN, Alrumaih AA (2021). Prevalence of irritable bowel syndrome and its associated risk factors among medical students in Riyadh, Saudi Arabia: A cross sectional study. Med Sci.

[25] Ibrahim NK, Battarjee WF, Almehmadi SA (2013). Prevalence and predictors of irritable bowel syndrome among medical students and interns in King Abdulaziz University, Jeddah. Libyan J Med.

[26] Ibrahim NK, Al-Bloushy RI, Sait SH, Al-Azhary HW, Al Bar NH, Mirdad GA (2016). Irritable bowel syndrome among nurses working in King Abdulaziz University Hospital, Jeddah, Saudi Arabia. Libyan J Med.

[27] Ibrahim NK, Al-Jamhoor SM, Ashor NM, Alsulami AA, Bukhari DA, Al-Bloshi AM, Fathi TT (2018). Irritable bowel syndrome among paramedical students, King Abdulaziz University, Jeddah, Saudi Arabia. J Adv Med Med Res.

[28] AlButaysh OF, AlQuraini AA, Almukhaitah AA, Alahmdi YM, Alharbi FS (2020). Epidemiology of irritable bowel syndrome and its associated factors in Saudi undergraduate students. Saudi J Gastroenterol.

[29] Alfaqih OY, Alnashri YM, Alharbi MM (2022). Prevalence and association between irritable bowel syndrome and stress among medical students in Al Qunfudah. Med Sci.

[30] Hakami RM, Elmakki E, Hasanain T (2019). Irritable bowel syndrome: Assessment of prevalence and risk factors in Saudi University students using Rome IV criteria. Gastroenterol Insights.

[31] Abbas S, Alotaibi A, Aldawsari AA (2017). The prevalence of irritable bowel syndrome (ibs) among male medical students in Majmaah University, Saudi Arabia. Int J Med Res Prof.

[32] Ahmed SA, Alotaibi YM, Alayed SI, Al Alshaykh OM, Alothman OM, Alhumaid AA (2020). Prevalence of irritable bowel syndrome among medical students in Imam Mohammad Ibn Saud Islamic University. Int J Med Dev Ctries.

[33] Al-bukhari I, Al-Malki K, Kashkari M, Alrifai A, Adnan M (2016). Prevalence and factors affecting irritable bowel syndrome among medical students at Taibah University. Clin Med Res.

[34] Alharbi SH, Alateeq FA, Alshammari KI (2019). Irritable bowel syndrome and dietary habits in Northern Saudi Arabia. Health.

[35] Alzahrani TA, Aljuaid AS, Alharthi TM, Aljabir AS, Alshehri LAA, Mahfouz ME, Masoodi I (2018). The prevalence and risk factors of irritable bowel syndrome among medical students and interns: results of a national survey in Saudi Arabia. Int J Med Health Res.

[36] AlAmeel T, Roth LS, Al Sulais E (2020). The prevalence of irritable bowel syndrome among board-certified medical doctors in Saudi Arabia: a cross-sectional study. J Can Assoc Gastroenterol.

[37] Sachithananthan V (2018). A study on the prevalence of irritable bowel syndrome (IBS) in Abha, Saudi Arabia. J Nutr Diet.

[38] Mohamed N, Khalid H, Abdullah K, Ramadan N, Alzuhairi R, Alqurashi R, El hasnaoui-Saadani R (2019). Irritable bowel syndrome among female students in Princess Nourah University in Kingdom of Saudi Arabia. Int J Sci: Basic Appl Res.

[39] Basharat V, Alsubaiei AM, Alshehri AZA (2022). Irritable bowel syndrome: prevalence, risk factor among Saudi population. Bahrain Medical Bull.

[40] Alsuwailm WA, Al-Qahtani MM, Al-Hulaibi AA, Al-Hadi MA, Busa'ad WT, Ali SI, Shehabeldeen SA (2017). Irritable bowel syndrome among medical students and interns in King Faisal University. Open J Prev Med.

[41] Alharbi W, Jahan S (2022). Prevalence and associated risk factors of irritable bowel syndrome among female secondary school students in Ar Rass City, Qassim Region. Health Psychol Res.

[42] Al-Turki Y, Aljulifi M, Al Murayshid A (2011). Prevalence of irritable bowel syndrome among students in King Saud University, Riyadh, Saudi Arabia. Middle East J Fam Med.

[43] Mirghani HO, Alshehri MM, Alotaibi JTA (2022). The prevalence of irritable bowel syndrome among medical students and interns at Tabuk University. Med Sci.

[44] Wani FA, Almaeen AH, Bandy AH (2020). Prevalence and risk factors of ibs among medical and nonmedical students in the Jouf University. Niger J Clin Pract.

[45] Almutairi M, AlQazlan M, Alshebromi A, Alawad M Prevalence of irritable bowel syndrome and its associated factors among medical students. Int J Med Res Health Sci.

[46] Alosaimi M, Ali A, Razzak HA (2016). Epidemiology of irritable bowel syndrome; a systematic review of literature. J Health Informatics Dev Ctries.

[47] Oka P, Parr H, Barberio B, Black C, Savarino E, Ford A (2020). Global prevalence of irritable bowel syndrome according to Rome III or IV criteria: a systematic review and meta-analysis. Lancet Gastroenterol Hepatol.

[48] Takeoka A, Kimura T, Hara S, Hamaguchi T, Fukudo S, Tayama J (2023). Prevalence of irritable bowel syndrome in Japan, China, and South Korea: an international cross-sectional study. J Neurogastroenterol Motil.

[49] Al-baghli N, Aleisa EM, Almoallem SH, Muhanna EH, Alkhawajah AA, Alherz HS (2022). Prevalence of irritable bowel syndrome in Saudi Arabia, a systematic review. Indo Am J P Sci.

[50] Creed F (2019). Review article: the incidence and risk factors for irritable bowel syndrome in population-based studies. Aliment Pharmacol Ther.

[51] Black CJ, Ford AC (2020). Global burden of irritable bowel syndrome: trends, predictions and risk factors. Nat Rev Gastroenterol Hepatol.

[52] Basharat V, Alsubaiei AM, Ali Alshehri AZ (2022). Irritable bowel syndrome: prevalence, risk factor among Saudi population. Bahrain Medical Bull.

[53] Black CJ, Craig O, Gracie DJ, Ford AC (2021). Comparison of the Rome IV criteria with the Rome III criteria for the diagnosis of irritable bowel syndrome in secondary care. Gut.

[54] Yang W, Yang X, Cai X (2022). The prevalence of irritable bowel syndrome among Chinese university students: a systematic review and meta-analysis. Front Public Health.

[55] Bashir Fadl AF, Al-Towerqi AM, Alharbi AA, Kabrah DK, Almalki AA, Algethami BN, Albogami AM (2022). Stress and a sedentary lifestyle are associated with irritable bowel syndrome in medical students from Saudi Arabia. Middle East J Fam Med.

[56] Vasquez-Rios G, Machicado JD, Ticse R (2019). Stress and a sedentary lifestyle are associated with irritable bowel syndrome in medical students from Peru: a cross-sectional study. Eur J Gastroenterol Hepatol.

[57] Chen Y, Lian B, Li P, Yao S, Hou Z (2022). Studies on irritable bowel syndrome associated with anxiety or depression in the last 20 years: a bibliometric analysis. Front Public Health.

[58] Liu H, Zou Y, Kan Y, Li X, Zhang Y (2022). Prevalence and influencing factors of irritable bowel syndrome in medical staff: a meta-analysis. Dig Dis Sci.

[59] Ibrahim NK (2016). A systematic review of the prevalence and risk factors of irritable bowel syndrome among medical students. Turk J Gastroenterol.

[60] Kim YS, Kim N (2018). Sex-gender differences in irritable bowel syndrome. J Neurogastroenterol Motil.

